# Supplementary material for: The Worldwide Prevalence of Internet Addiction among Medical Students: A Systematic Review and Meta-Analysis
Source: Int J Environ Res Public Health. 2024 Aug 29;21(9):1146. doi: 10.3390/ijerph21091146 (PMC11430859; doi:10.3390/ijerph21091146)
Supplement: Supplementary file 1 [file ijerph-21-01146-s001.zip › Table S2.pdf]

Table S2. JBI risk of bias quality assessment for prevalence studies.

study	Items on the Joanna Briggs Institute critical appraisal checklist for prevalence studies									score and %	risk of bias	overall appraisal
	Q1	Q2	Q3	Q4	Q5	Q6	Q7	Q8	Q9			
Krajewska-Kułłak	0	0	0	0	0	1	0	1	0	2/9 = 22.2%	high	exclude
et al. (2011) [1]	0	0	0	0	0	1	0	0	0	1/9 = 11.1%		
Ghamari et al.	0	1	0	1	0	1	0	1	0	4/9 = 44.4%	high	exclude
(2011) [2]	0	1	0	1	0	1	0	1	0	4/9 = 44.4%		
Berner et al.	0	0	0	1	0	1	0	1	1	4/9 = 44.4%	high	exclude
(2014)[3]	1	0	0	1	0	1	0	1	0	4/9 = 44.4%		
Srijampana et al.	0	1	0	0	0	1	0	1	0	3/9 = 33.3%	high	exclude
(2014) [4]	0	1	0	0	0	1	0	1	0	3/9 = 33.3%		
Capetillo-Ventura	1	1	1	1	0	1	0	1	0	6/9 = 66.6%	moderate	include
& Juárez-Treviño (2015) [5]	1	1	1	1	0	1	0	1	0	6/9 = 66.6%		
Chaudhari et al.	1	1	0	1	0	1	0	1	1	6/9 = 66.6%	moderate	include
(2015)[6]	0	1	0	1	1	1	0	1	1	6/9 = 66.6%		
Mohammadbeigi	1	1	0	1	0	1	0	1	0	5/9 = 55.5%	moderate	include
et al. (2016)[7]	0	1	0	1	0	1	0	1	0	4/9 = 44%	high	exclude/ include (final decision)
Shaheen &	1	1	1	1	0	1	0	1	1	7/9 = 77%	low	include
Farahat (2016) [8]	1	1	1	1	0	1	0	1	1	7/9 = 77%		
Ali et al. (2017)	1	1	1	1	1	1	1	1	1	9/9 = 100%	low	include
[9]	1	1	1	1	1	1	1	1	1	9/9 = 100%		
Ranganatha &	0	0	1	0	0	1	0	0	0	2/9 = 22.2%	high	exclude
Usha (2017) [10]	0	0	1	0	1	0	0	0	0	2/9 = 22.2%		
Ahmer & Tanzil	0	0	1	0	1	1	0	1	0	4/9 = 44.4%	high	exclude
(2018)[11]	0	0	1	0	0	1	0	0	1	3/9 = 33.3%		
Haroon et al.	0	1	1	1	1	1	0	1	1	7/9 = 77%	low	include
(2018)[12]	0	1	1	1	1	1	0	1	1	7/9 = 77%		
Taha et al. (2019)	0	1	1	1	0	1	1	1	1	7/9 = 77%	low	include
[13]	1	1	1	1	1	1	1	1	1	9/9 = 100%		
Javaeed et al.	1	1	1	1	0	1	1	1	1	8/9 = 88.8%	low	include
(2019) [14]	1	1	1	1	0	1	1	1	1	8/9 = 88.8%		
Javaeed et al.	0	0	0	1	0	1	0	1	1	4/9 = 44.4%	high	exclude
(2020) [15]	0	0	0	1	0	1	0	1	1	4/9 = 44.4%		
Mboya et al.	0	1	1	1	1	1	1	1	0	7/9 = 77%	low	include
(2020) [16]	0	1	1	1	1	1	1	1	0	7/9 = 77%		
Kolaib et al.	0	0	0	1	0	1	0	1	0	3/9 = 33.3%	high	exclude
(2020) [17]	0	0	0	1	0	1	0	1	0	3/9 = 33.3%		
Dhamnetiya et al.	1	1	1	1	0	1	0	1	1	7/9 = 77%	low	include
(2021) [18]	0	1	1	1	1	1	1	1	1	8/9 = 88.8%		
Ibrahim et al.	1	1	1	1	1	1	1	1	1	9/9 = 100%	low	include
(2022) [19]	0	1	1	1	1	1	0	1	1	7/9 = 77%		
Chauhan et al.	1	1	1	1	0	1	0	1	0	6/9 = 66.6%	moderate	include
(2022) [20]	1	1	1	1	0	1	0	1	0	6/9 = 66.6%		

Note. The comparison of JBI ratings was given by Z.S. and Z.K. Q1 to Q9 represent the questions of the JBI critical appraisal checklist.

References

1. Krajewska-Kułak, E.; Kułak, W.; Marcinkowski, J.T.; Damme-Ostapowicz, K.V.; Lewko, J.; Lankau, A.; Łukaszuk, C.; Rozwadowska, E. Internet Addiction Among Students of the Medical University of Białystok. *CIN Comput. Inform. Nurs.* **2011**, *29*, 657–661, doi:10.1097/NCN.0b013e318224b34f.
2. Ghamari, F.; Mohammadbeigi, A.; Mohammadsalehi, N.; Hashiani, A.A. Internet Addiction and Modeling Its Risk Factors in Medical Students, Iran. *Indian J. Psychol. Med.* **2011**, *33*, 158–162, doi:10.4103/0253-7176.92068.
3. Berner, J.E.; Santander, J.; Contreras, A.M.; Gómez, T. Description of Internet Addiction among Chilean Medical Students: A Cross-Sectional Study. *Acad. Psychiatry* **2014**, *38*, 11–14, doi:10.1007/s40596-013-0022-6.
4. Srijampana, V.V.G.R.; Endreddy, A.R.; Prabhath, K.; Rajana, B. Prevalence and Patterns of Internet Addiction among Medical Students. *Med. J. Dr Patil Univ.* **2014**, *7*, 709–713, doi:10.4103/0975-2870.144851.
5. Capetillo-Ventura, N.; Juárez-Treviño, M. Internet Addiction in University Medical Students. *Med. Univ.* **2015**, *17*, 88–93, doi:10.1016/j.rmu.2015.02.003.
6. Chaudhari, B.; Menon, P.; Saldanha, D.; Tewari, A.; Bhattacharya, L. Internet Addiction and Its Determinants among Medical Students. *Ind. Psychiatry J.* **2015**, *24*, 158–162, doi:10.4103/0972-6748.181729.
7. Mohammadbeigi, A.; Valizadeh, F.; Mirshojaee, S.R.; Ahmadli, R.; Mokhtari, M.; Ghaderi, E.; Ahmadi, A.; Rezaei, H.; Ansari, H. Self-Rated Health and Internet Addiction in Iranian Medical Sciences Students; Prevalence, Risk Factors and Complications. *Int. J. Biomed. Sci. IJBS* **2016**, *12*, 65–70.
8. Shaheen, H.M.; Farahat, T.M. Problematic Internet Use among Medical School Students in Menoufia University Egypt. *J. Child Adolesc. Behav.* **2016**, *4*, 1–5, doi:10.4172/2375-4494.1000298.
9. Ali, R.; Mohammed, N.; Aly, H. Internet Addiction among Medical Students of Sohag University, Egypt. *J. Egypt. Public Health Assoc.* **2017**, *92*, 86–95, doi:10.21608/EPX.2018.8946.
10. Ranganatha, S.; Usha, S. Prevalence and Pattern of Internet Addiction among Medical Students, Bengaluru. *Int. J. Community Med. Public Health* **2017**, *4*, Article 4680, doi:10.18203/2394-6040.ijcmph20175350.
11. Ahmer, Z.; Tanzil, S. Internet Addiction among Social Networking Sites Users: Emerging Mental Health Concern among Medical Undergraduates of Karachi. *Pak. J. Med. Sci.* **2018**, *34*, 1473–1477, doi:10.12669/pjms.346.15809.
12. Haroon, M.Z.; Zeb, Z.; Javed, Z.; Awan, Z.; Aftab, Z.; Talat, W. Internet Addiction in Medical Students. *J. Ayub Med. Coll. Abbottabad JAMC* **2018**, *30*, S659–S663.
13. Taha, M.H.; Shehzad, K.; Alamro, A.S.; Wadi, M. Internet Use and Addiction Among Medical Students in Qassim University, Saudi Arabia. *Sultan Qaboos Univ. Med. J. SQUMJ* **2019**, *19*, e142–e147, doi:10.18295/squmj.2019.19.02.010.
14. Javaeed, A.; Zafar, M.B.; Iqbal, M.; Ghauri, S.K. Correlation between Internet Addiction, Depression, Anxiety and Stress among Undergraduate Medical Students in Azad Kashmir. *Pak. J. Med. Sci.* **2019**, *35*, 506–509, doi:10.12669/pjms.35.2.169.
15. Javaeed, A.; Jeelani, R.; Gulab, S.; Ghauri, S.K. Relationship between Internet Addiction and Academic Performance of Undergraduate Medical Students of Azad Kashmir. *Pak. J. Med. Sci.* **2020**, *36*, 229–233, doi:10.12669/pjms.36.2.1061.
16. Mboya, I.B.; Leyaro, B.J.; Kongo, A.; Mkombe, C.; Kyando, E.; George, J. Internet Addiction and Associated Factors among Medical and Allied Health Sciences Students in Northern Tanzania: A Cross-Sectional Study. *BMC Psychol.* **2020**, *8*, 1–8, doi:10.1186/s40359-020-00439-9.
17. Kolaib, A.A.; Alhazmi, A.H.; Kulaib, M.A. Prevalence of Internet Addiction and Its Associated Factors among Medical Students at Taiba University, Saudi Arabia. *J. Fam. Med. Prim. Care* **2020**, *9*, 4797–4800, doi:10.4103/jfmpc.jfmpc_655_20.
18. Dhamnetiya, D.; Singh, S.; Jha, R.P. Correlates of Problematic Internet Use among Undergraduate Medical Students of Delhi. *BMC Psychiatry* **2021**, *21*, 1–8, doi:10.1186/s12888-021-03529-z.
19. Ibrahim, A.K.; Fouad, I.; Kelly, S.J.; El Fawal, B.; Ahmed, G.K. Prevalence and Determinants of Internet Addiction among Medical Students and Its Association with Depression. *J. Affect. Disord.* **2022**, *314*, 94–102, doi:10.1016/j.jad.2022.07.007.
20. Chauhan, N.; Tiwari, P.; Ahlawat, P.; Singh, S.K.; Kamble, B.D.; Mahaur, G. Internet Addiction and Sleep Quality among Medical Students of Delhi: A New Age Epidemic. *Natl. J. Community Med.* **2022**, *13*, 864–868, doi:10.55489/njcm.131220222488.
