# Supplementary figures and images for: The Worldwide Prevalence of Internet Addiction among Medical Students: A Systematic Review and Meta-Analysis
Source: Int J Environ Res Public Health. 2024 Aug 29;21(9):1146. doi: 10.3390/ijerph21091146 (PMC11430859; doi:10.3390/ijerph21091146)

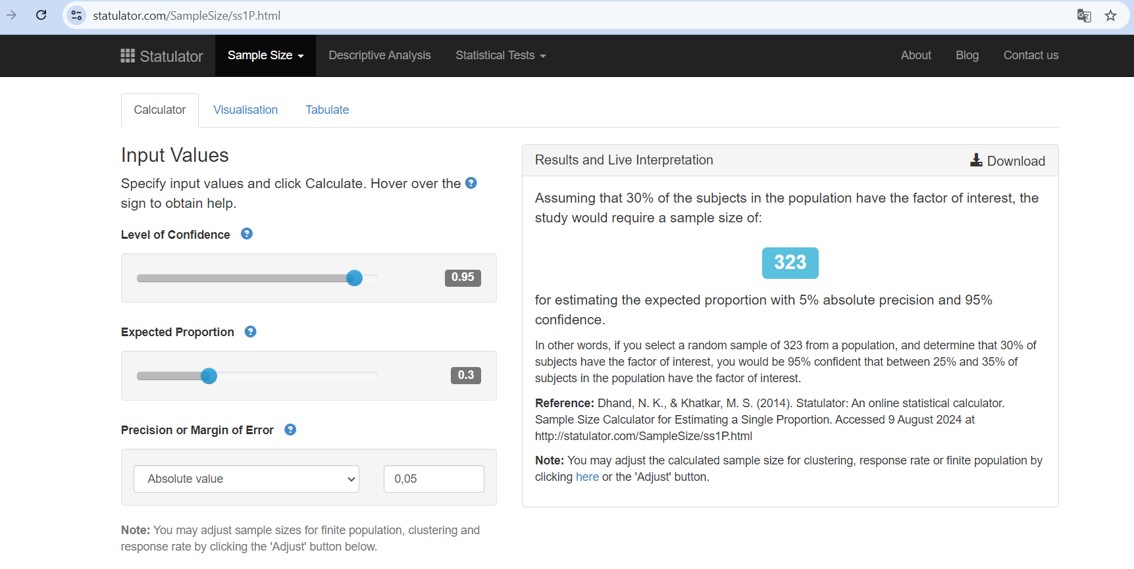

Supplement: Supplementary file 1 [file ijerph-21-01146-s001.zip › Figure S1.jpg]

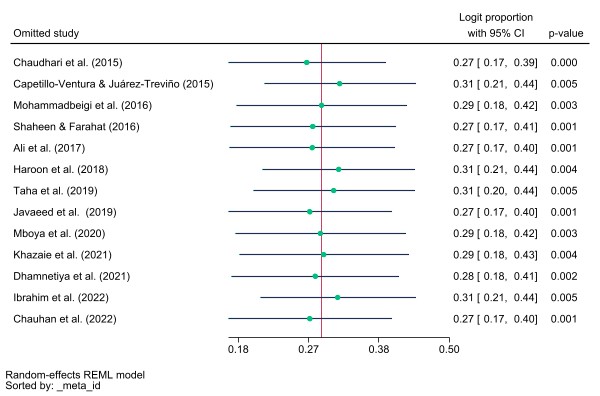

Supplement: Supplementary file 1 [file ijerph-21-01146-s001.zip › Figure S2.jpg]

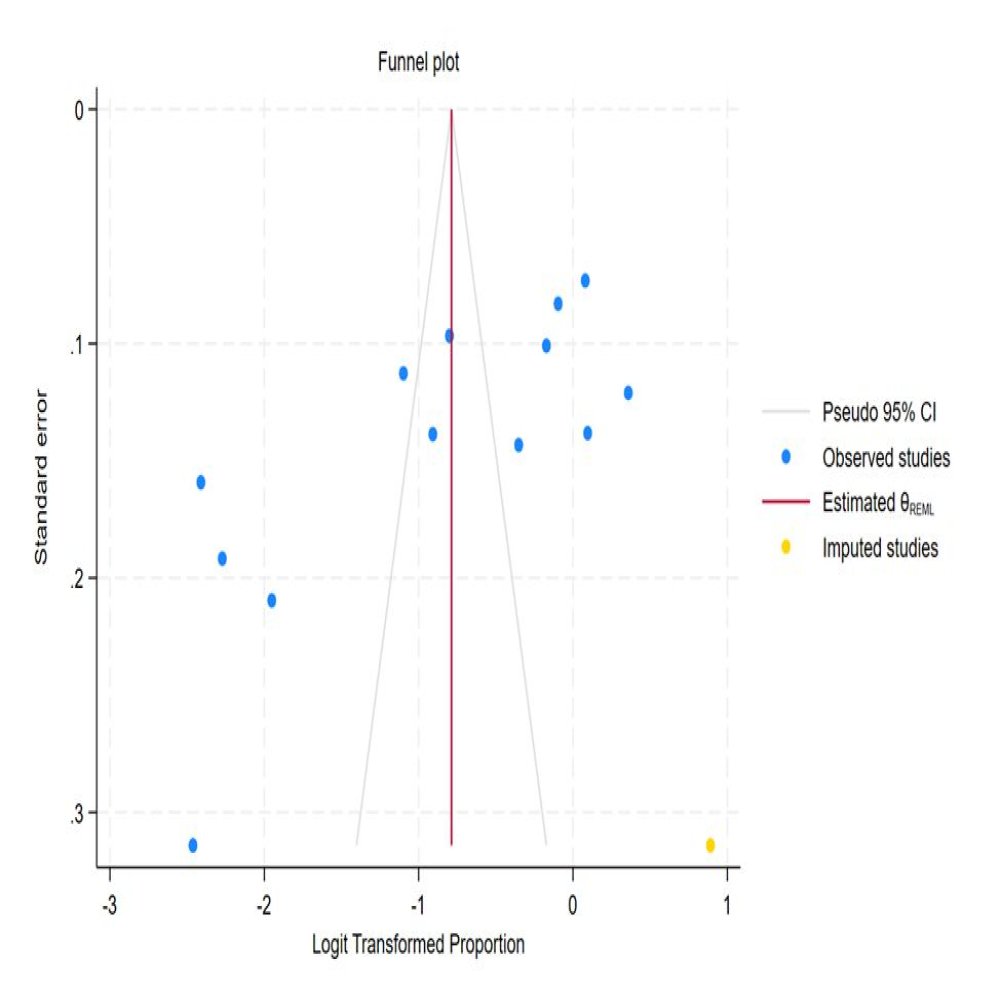

Supplement: Supplementary file 1 [file ijerph-21-01146-s001.zip › Figure S3.jpg]
